# In Mitochondria β-Actin Regulates mtDNA Transcription and Is Required for Mitochondrial Quality Control

**DOI:** 10.1016/j.isci.2018.04.021

**Published:** 2018-05-03

**Authors:** Xin Xie, Tomas Venit, Nizar Drou, Piergiorgio Percipalle

**Affiliations:** 1 Science Division, Biology Program, New York University Abu Dhabi (NYUAD), P.O. Box 129188, Abu Dhabi, United Arab Emirates; 2 NYU Abu Dhabi Center for Genomics and Systems Biology, Abu Dhabi, UAE; 3 Department of Molecular Biosciences, The Wenner-Gren Institute, Stockholm University, S-106 91 Stockholm, Sweden

**Keywords:** Molecular Biology, Cell Biology, Functional Aspects of Cell Biology

## Abstract

In eukaryotic cells, actin regulates both cytoplasmic and nuclear functions. However, whether actin-based structures are present in the mitochondria and are involved in mitochondrial functions has not been investigated. Here, using wild-type β-actin +/+ and knockout (KO) β-actin −/− mouse embryonic fibroblasts we show evidence for the defect in maintaining mitochondrial membrane potential (MMP) in β-actin-null cells. MMP defects were associated with impaired mitochondrial DNA (mtDNA) transcription and nuclear oxidative phosphorylation (OXPHOS) gene expression. Using super-resolution microscopy we provided direct evidence on the presence of β-actin-containing structures inside mitochondria. Large aggregates of TFAM-stained nucleoids were observed in bulb-shaped mitochondria in KO cells, suggesting defects in mitochondrial nucleoid segregation without β-actin. The observation that mitochondria-targeted β-actin rescued mtDNA transcription and MMP suggests an indispensable functional role of a mitochondrial β-actin pool necessary for mitochondrial quality control.

## Highlights

• Maintenance of mitochondrial membrane potential (MMP) requires β-actin
• MMP defects are due to impaired mtDNA and nucleus-encoded OXPHOS gene transcription
• Loss of mitochondrial β-actin-containing structures leads to nucleoid aggregation
• Mitochondria-targeted β-actin rescued mtDNA transcription and MMP

## Graphical Abstract

**Figure:**
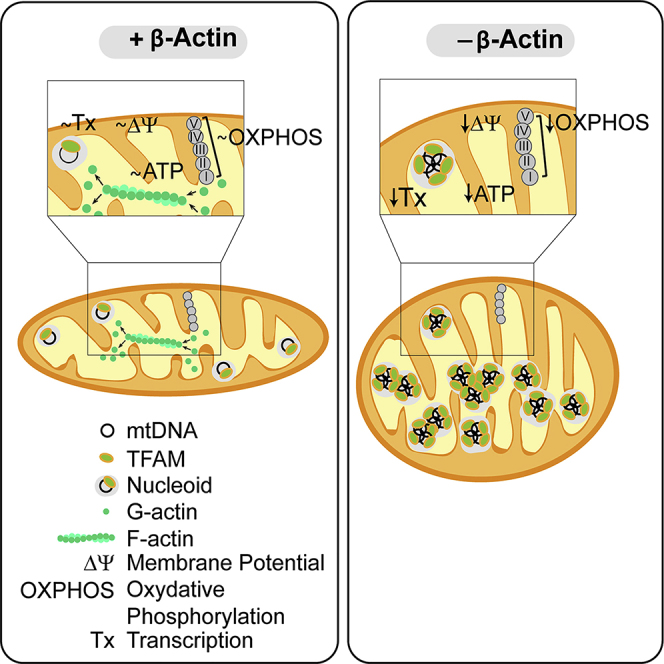

## Introduction

In the cytoplasm actin is known to regulate cell morphology, movement, and organelle dynamics and function (Dominguez and Holmes, 2011). Specific interactions of cytosolic actin with mitochondria are known to mediate fission of mitochondrial networks and mitochondrial transport, contributing to cellular distribution of mitochondria (Boldogh and Pon, 2006, Senning and Marcus, 2010). Although the exact mechanism remains to be fully unveiled, cytosolic actin is also involved in mitochondria-dependent apoptosis (Gourlay and Ayscough, 2005). Evidence also suggests that actin and some actin-associated proteins such as myosin are involved in mitochondrial function through specific association with mitochondrial DNA (mtDNA) (Reyes et al., 2011).

Functional association of actin with DNA has been described in both prokaryotes and eukaryotes (Moller-Jensen et al., 2002, Visa and Percipalle, 2010). In the eukaryotic cell nucleus, actin interacts with active genes and controls transcription by all three eukaryotic nuclear RNA polymerases (Visa and Percipalle, 2010). At the genomic level, β-actin regulates chromatin distribution and deposition of epigenetic marks, leading to activation or repression of gene programs, and affects cellular identity (Xie et al., 2018). In rod-shaped bacteria, actin-like proteins play an important role in genomic and plasmid DNA segregation (Kruse and Gerdes, 2005, Moller-Jensen et al., 2002). Given their circular genome and the fact that mtDNA maintenance and replication are independently performed, it is possible that actin-based mechanisms are fundamental for mtDNA segregation. Indeed, β-actin and myosin were found to associate with mitochondrial nucleoids, and a pool of β-actin resistant to protease digestion was identified in isolated mitochondria (Reyes et al., 2011), suggesting that β-actin is localized in mitochondria. However, how β-actin is organized inside mitochondria and whether mitochondrial β-actin plays a functional role in mitochondrial quality control is unknown.

In this study, we analyzed embryonic fibroblasts from wild-type (WT) β-actin +/+ mice and knockout (KO) β-actin −/− mice (Tondeleir et al., 2012). When comparing KO cells lacking both functional β-actin alleles with WT cells, we found a severe defect in maintaining mitochondrial membrane potential (MMP). This defect can be attributed to impaired mtDNA transcription and down-regulation of nucleus-encoded oxidative phosphorylation (OXPHOS) genes. Using super-resolution microscopy, we observed β-actin-containing structures inside mitochondria, which seem to be connected to the cytosolic counterparts. In the absence of β-actin, mitochondrial nucleoids tend to form large aggregates in bulbous mitochondria. Together with the significantly increased mtDNA copy number in KO cells, our finding suggests that mitochondrial nucleoids are defective in segregation after mtDNA replication. Importantly, mitochondria-targeted β-actin rescued mtDNA transcription and MMP when constitutively re-introduced into β-actin-null cells. Overall, our study provides direct evidence on the presence of β-actin in mitochondria and unveils a yet unknown role of the mitochondrial pool of β-actin in mitochondrial quality control.

## Results

### Defects of Mitochondrial Membrane Potential (MMP) Maintenance in Cells Lacking β-Actin

**Figure 1:**
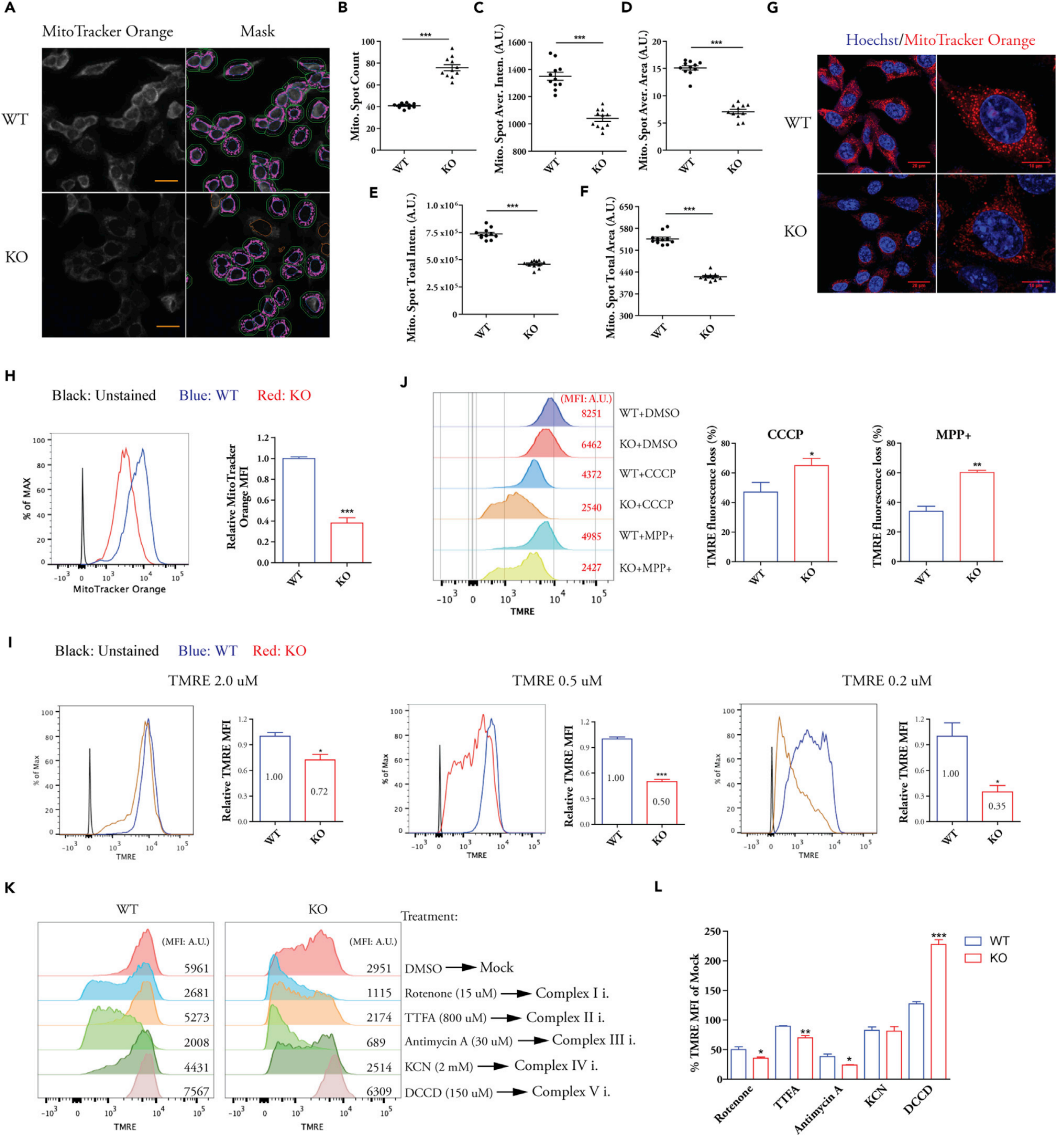
Impaired MMP and Hypersensitivity to Mitochondrial Stress in β-Actin Knockouts (A) WT and KO MEFs stained with MitoTracker Orange were analyzed using a high-content phenotypic platform. Right panel shows the mask of detected staining signal. The green color delineates the simulated cytoplasmic boundary of individual cells, and the inner blue circle defines the nuclear region of stained DNA. The magenta color displays the detected MitoTracker Orange staining within the simulated cytoplasm. Scale bar, 20 μm. (B–F) Quantification of MitoTracker Orange signal in single cells. (B) Spot count; (C) spot average intensity; (D) spot average area; (E) spot total intensity; and (F) spot total area. Each data point represents mean of at least 500 single cells in one biological replicate, representative of three independent experiments. (G) Confocal image of MEFs stained with MitoTracker Orange. (H) FACS analysis of MEFs stained with MitoTracker Orange. Data are the summary of the mean fluorescence intensity (MFI), n = 3 independent experiments. (I) FACS analysis of MEFs stained with decreasing concentrations of TMRE. The relative TMRE MFI of WT cells and KO cells at each condition was calculated; n = 3 independent experiments. (J) FACS analysis of MMP change after MPP+ (2 mM) and CCCP (30 μM) treatment by TMRE staining; n = 3 independent experiments. DMSO treatment was used as mock control to calculate the % TMRE fluorescence loss. DMSO, dimethyl sulfoxide. (K and L) MEFs were treated with selective inhibitors of complex I to V. MMP changes were analyzed by TMRE staining (K). Mean TMRE fluorescence intensity change relative to mock is shown in (L); n = 3 biological replicates. Data are presented as mean ± SEM *p ≤ 0.05, **p ≤ 0.01, ***p ≤ 0.001. Student's t test. SEM, standard error of the mean. See also Figure S1 and Videos S1, S2, and S3.

We used high-content screening platform to analyze the mitochondrial features of mouse embryonic fibroblasts from WT (β-actin +/+) and KO (β-actin −/−) embryos (Tondeleir et al., 2012). Cells stained by a MitoTracker Orange dye, which accumulates in the mitochondria depending on the membrane potential and is well retained after fixation, were subjected to quantitative analysis of stained mitochondrial spots (MitoTracker Orange-positive staining) in single cells (Figure 1A). We found that, in comparison with WT cells, there were more stained spots in the cytoplasm of KO cells (Figure 1B). However, the average spot intensity is significantly lower and the average spot area is smaller in KO cells (Figures 1C and 1D). The value of total spot area and intensity in each cell was also significantly lower in KO cells on average (Figures 1E and 1F). These data indicate that, in the absence of β-actin, smaller mitochondrial regions could maintain membrane potential, leading to an overall lower MMP in KO cells. Confocal images essentially confirmed the results from the high-content screening (Figure 1G). Fluorescence-activated cell sorting (FACS) analysis demonstrated that KO cells lost more than 50% MMP based on MitoTracker Orange staining (Figure 1H). The significant loss of MMP was not observed in heterozygous cells with only one functional β-actin allele (Figure S1A). Therefore, the severe MMP loss only happens in the absence of two functional β-actin alleles.

We further used tetramethylrhodamine ethyl ester (TMRE) that dynamically accumulates or associates with mitochondria depending on MMP to stain living cells. The staining intensity declined with decreasing concentrations of TMRE applied to both WT and KO cells. However, at all concentrations, KO cells displayed significantly lower TMRE staining intensity compared with WT cells (Figure 1I). Remarkably, the difference of TMRE intensity between WT and KO cells became larger at lower concentration (Figure 1I). Therefore, KO cells displayed defects in accumulating TMRE especially at low concentration, indicating impaired ability to maintain the dynamic MMP.

### Increased Sensitivity to Mitochondrial Stress and Altered Mitochondrial Morphology in the Absence of β-Actin

We next investigated MMP changes under mitochondrial stress induced by MPP+ (1-methyl-4-phenylpyridinium, a specific complex I inhibitor) and CCCP (Carbonyl cyanide m-chlorophenyl hydrazone, a mitochondrial uncoupler) (Qi et al., 2013). MPP+ and CCCP treatment reduced TMRE staining in both WT and KO cells (Figure 1J, left panel). However, KO cells lost more MMP (TMRE intensity) after MPP+ and CCCP stress treatment (Figure 1J, right panel). Consistently, live cell images demonstrated that KO cells are very sensitive to CCCP stress and display rapid cell shrinkage after CCCP addition (Video S1). Furthermore, after mitochondrial membrane depolarization by high concentration of CCCP, KO cells also showed an impaired recovery of MMP compared with WT cells (Figures S1B and S1C). Together, these data show that cells without β-actin are more sensitive to mitochondrial stress caused by MMP impairment, which in turn is linked to an intrinsic defect in MMP maintenance.

We further applied selective inhibitors of electron transport chain complexes and found that KO cells are more sensitive to the activity inhibition of complexes I, II, and III, as manifested by the greater loss of MMP when compared with WT cells (Figures 1K and 1L). Importantly, inhibition of complex V (ATP synthase) by DCCD (*N*,*N*-dicyclohexylcarbodiimide) led to MMP increase in both WT and KO cells; however, the degree of MMP increase is much higher in KO cells (Figure 1L). Since DCCD inhibits proton translocation in ATP synthase (complex V) without significant effect on electron transfer activity (Clejan et al., 1984, Toei and Noji, 2013), our results suggest that the mitochondrial intermembrane space has more unused capacity for proton storage in KO cells than in WT cells when ATP synthase is blocked. Therefore, the lower MMP in KO cells is due to the lack of proton accumulation instead of the lack of proton storage capacity of mitochondria.

Several studies showed that MMP alterations could lead to changes in mitochondrial morphology (Leonard et al., 2015, Safiulina et al., 2006). We therefore investigated mitochondrial morphology in live cells using MitoTracker Deep Red, which accumulates in mitochondria regardless of the MMP. As expected, we found that the majority of WT cells showed a network of thin filamentous mitochondria. In contrast, KO cells exhibited swollen mitochondria with bulbous shape (Figure S1D and Video S2). Since mitochondria swelling and increase in volume are associated with loss of MMP (Leonard et al., 2015, Safiulina et al., 2006), the observed mitochondrial morphology in KO cells is likely to be caused by the severely impaired MMP. We further tested whether reducing MMP in WT cells leads to similar morphological changes. In the presence of MPP+, we observed the gradual formation of bulbous mitochondria after 30-min treatment in WT cells (Figure S1E). Live cell images showed that the initially rod-shaped mitochondria gradually shortened along the length and became spherical (Video S3). The level of bulbous mitochondria was also found to increase in KO cells after MPP+ treatment (Video S3). Collectively, our data show that β-actin is indispensable in controlling MMP and the morphology of mitochondria.

### In β-Actin Knockouts, Impaired Transcription of OXPHOS Genes from mtDNA and Nucleus Is Coupled to Decreased OXPHOS Activity

**Figure 2:**
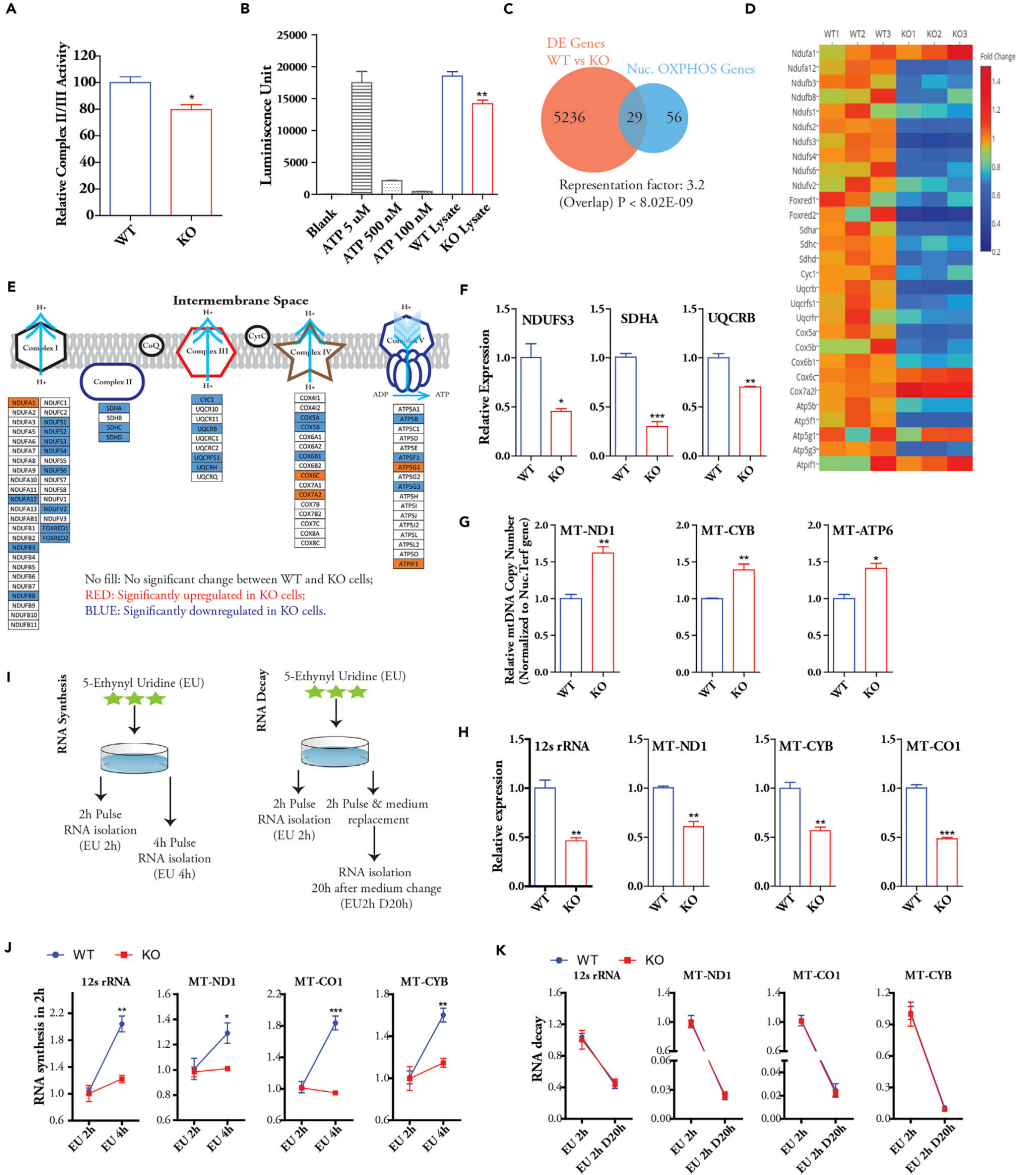
OXPHOS Defects Are Linked to Impaired mtDNA and Nuclear OXPHOS Gene Expression in β-Actin Knockouts (A) Mito complex II/III activity assay of isolated mitochondria; n = 3 independent experiments. (B) Cellular ATP level determination; n = 3 independent experiments. (C) Venn diagram showing significant overlap of differentially expressed genes in WT versus KO MEFs and OXPHOS genes encoded by nucleus: Fisher's exact test. (D) Relative expression levels of nuclear OXPHOS genes differentially expressed between WT and KO cells by RNA-seq. The mean value of WT samples was set as 1. (E) Distribution of differentially expressed nuclear OXPHOS genes. (F) Quantification of mRNA level of Ndufs3, Sdha, and Uqcrb genes by qPCR; n = 3 biological replicates. (G) mtDNA level determination using MT-ND1, MT-CYB, and MT-ATP6 genes by qPCR; n = 3 independent experiments. (H) Transcript level of 12s rRNA, MT-ND1, MT-CYB, and MT-CO1 genes by qPCR; n = 3 independent experiments. (I–K) Schematics of RNA synthesis and RNA decay experiments are shown in (I). qPCR analysis of mt-RNA synthesis (J) and decay (K); n = 3 biological replicates. Data are presented as mean ± SEM *p ≤ 0.05, **p ≤ 0.01, ***p ≤ 0.001. Student's t-test. SEM, standard error of the mean. See also Figure S2.

MMP is maintained by the activity of electron transport chain complexes during OXPHOS (Dzbek and Korzeniewski, 2008). To study the electron transport activity in the absence of β-actin, we isolated mitochondria from WT and KO cells and compared the complex II/III activity. Mitochondria from KO cells showed a significantly lower level of complex II/III activity (Figure 2A). Consistently, KO cells produced an overall lower cellular ATP level than WT cells (Figure 2B), altogether demonstrating an impaired OXPHOS activity in the absence of β-actin. We then analyzed the relative expression levels of nuclear OXPHOS genes using a recently published RNA-seq dataset (Xie et al., 2018). Remarkably, nuclear OXPHOS genes were found to be significantly overrepresented in the differentially expressed (DE) genes between WT and KO cells (Figure 2C). These results imply that impaired OXPHOS activity in KO cells is a direct consequence of altered OXPHOS gene expression. Consistent with impaired MMP, the majority of DE OXPHOS genes showed down-regulation in KO cells (Figure 2D). These down-regulated genes mainly encode components of complexes I, II, and III in the electron transport chain (Figure 2E). We verified the lower expression of *Ndufs3*, *Sdha,* and *Uqcrb* in KO cells by quantitative polymerase chain reaction (qPCR) (Figure 2F). The overall down-regulation tendency of OXPHOS genes is consistent with the previous finding that nuclear components of OXPHOS are transcriptionally co-regulated (van Waveren and Moraes, 2008).

Essential components of electron transport complexes are also encoded by the mitochondrial genome. There is evidence that β-actin and myosin play a role in mtDNA topology and copy number maintenance (Reyes et al., 2011). However, how the mtDNA copy number and mtDNA transcription are potentially regulated by β-actin is not known. We therefore analyzed the status of mtDNA copy number and its transcript in KO cells. We found that in the absence of β-actin there is an increase in mtDNA copy number, as revealed by the quantification of a subset of mtDNA loci (Figure 2G). Furthermore, total DNA sequencing also revealed an overall higher mtDNA level across the mtDNA genome in KO cells (Figure S2A). However, the expression of transcripts from mtDNA was significantly reduced in KO cells (Figure 2H). The decreased level of mtDNA transcript may be due to decreased RNA synthesis or increased RNA degradation. To distinguish between the two possibilities, newly synthesized RNA was labeled with 5-ethynyl uridine to monitor the rate of RNA synthesis and decay (Figure 2I, see also Transparent Methods). For RNA synthesis, we pulse-labeled the cells for 2 and 4 hr to quantify the relative RNA changes within 2-hr intervals (Figure 2I, left panel). For the RNA decay experiment, labeled cells were cultured in fresh medium to determine the remaining amount of labeled RNA after 20 hr (Figure 2I, right panel). We observed that the absence of β-actin in KO cells leads to impaired RNA synthesis without affecting the RNA decay (Figures 2J and 2K). Altogether, our data demonstrate that in the absence of β-actin, nuclear OXPHOS genes are down-regulated and mtDNA transcription is impaired, suggesting that nuclear and mitochondrial genomes are coordinated to balance the expression of OXPHOS components. The correlation between nuclear and mitochondrial expression of OXPHOS subunits has also been reported in previous studies (Gagnon et al., 1991, Murdock et al., 1999). Consistent with reduced expression of OXPHOS components, KO cells displayed an overall lower mitochondrial mass as assessed by MitoTracker Deep Red staining (Figure S2B) (Schieke et al., 2006).

### A Pool of β-Actin Is Located Inside Mitochondria and Mitochondrial Nucleoids form Large Aggregates in the Absence of β-Actin

As both nuclear OXPHOS genes and mtDNA transcription are affected without β-actin, a key question is whether β-actin functions primarily in the cytoplasm or in the nucleus to control OXPHOS genes. We first used the recently published plasmids containing green fluorescent protein (GFP)-β-actin and GFP-nuclear localization signal (NLS)-β-actin (Sharili et al., 2016) and transiently expressed them in KO cells. As expected, the GFP-β-actin was mainly in the cytoplasm and the NLS-β-actin was enriched in the nucleus (Figure S3A). However, neither β-actin nor NLS-β-actin showed rescue effects on the MMP in KO cells (Figures S3B–S3D). The level of overexpressed exogenous β-actin in KO cells is much lower than that of the endogenous β-actin in WT cells (Figure S3E). Nevertheless, the fact that nucleus-targeted β-actin failed to rescue MMP prompted us to investigate a potential role of β-actin inside the mitochondria.

**Figure 3:**
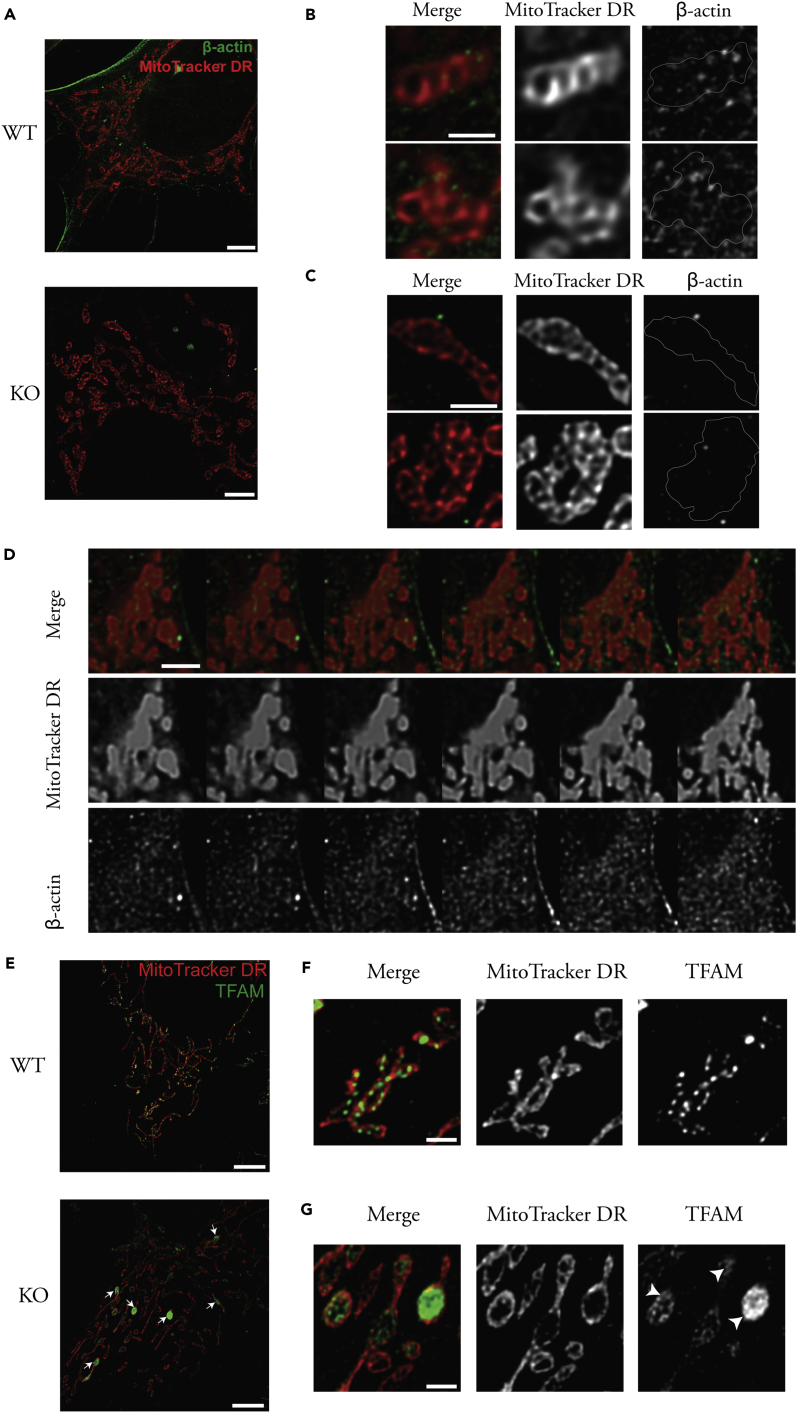
A Pool of β-Actin Resides in Mitochondria, Without Which TFAM-Stained Nucleoids Tend to Form Large Aggregates WT and KO MEFs were stained with MitoTracker Deep Red (red) and a β-actin-specific antibody (green) or anti-TFAM antibody (green) and visualized by STED microscope. (A) Comparison of β-actin staining and its distribution between WT and KO cells. Scale bar, 5  μm. (B and C) STED microscope image insets selected from WT cell (B) and KO cell (C). Gray lines represents the boundary of mitochondria in the β-actin insets; scale bar, 1  μm. (D) Montage of six consecutive z stack confocal images in WT cells, moving 250 nm in each step. Scale bar, 2  μm. (E) STED microscope image of MitoTracker Deep Red (red) and anti-TFAM antibody (green) staining in WT and KO cells; scale bar, 5  μm. Arrows point to examples of enlarged mitochondria. (F and G) STED microscope image insets selected from WT cell (F) and KO cell (G); scale bar, 1  μm; arrowheads in (G) indicate TFAM-based nucleoid aggregates. See also Figures S3 and S4.

A previous report showed that mtDNA associates with β-actin and myosin, and a pool of β-actin in isolated mitochondria seems to be resistant to protease digestion (Reyes et al., 2011, Wang and Bogenhagen, 2006). However, there is no direct evidence showing that β-actin-containing structures reside in the mitochondria of mammalian cells. To demonstrate the localization of β-actin inside mitochondria, we co-stained WT and KO MEFs with MitoTracker Deep Red and β-actin-specific antibody, and visualized the cells by stimulated emission depletion (STED) microscopy. Consistent with a previous study (Shestakova et al., 2001), β-actin was enriched at the plasma membrane in WT cells, with weaker staining of β-actin-containing structures in the cytoplasm (Figure 3A). The absence of β-actin staining in KO cells demonstrate the specificity of the β-actin antibody (Figure 3A). Detailed analysis showed that β-actin-containing structures were widely distributed in both the cytoplasm and mitochondria in WT cells, which are absent in KO cells (Figures 3B and 3C). It is noteworthy that β-actin seems to form an interconnected network in both the cytoplasm and mitochondria of WT cells (Figure 3B). z Stack confocal images also showed that β-actin is localized around as well as inside the mitochondria in WT cells (Figure 3D). Together, these results provide strong evidence that β-actin resides in the mitochondria and seems to be connected with cytoplasmic counterparts.

We also stained WT cell with phalloidin, which selectively binds to polymerized F-actin. The strongest phalloidin staining localized at the cell periphery or the actin fibers inside cytoplasm (Figure S4A). Phalloidin staining was observed around the mitochondria, and a relatively weak and network-like phalloidin staining was also seen inside the mitochondria and in the cytoplasm (Figure S4B). The data therefore suggest that, in addition to forming strong actin fibers, polymerized actins form network-like structures both around and inside the mitochondria. However, it should be pointed out that phalloidin stains F-actins formed by different actin isoforms.

The presence of β-actin network inside mitochondria resembles actin-like cytoskeleton in bacteria, which is essential for plasmid partitioning (Carballido-Lopez, 2006, Kruse and Gerdes, 2005). We then analyzed the nucleoid based on mitochondrial transcription factor A (TFAM) staining, which is the main factor for mtDNA packaging (Kukat and Larsson, 2013). In WT mitochondria, we observed a relatively uniform size of TFAM-based nucleoid, which is efficiently distributed along the tubular mitochondria (Figures 3E and 3F). This is consistent with recent studies in which mammalian mitochondrial nucleoids show uniform size and frequently contain a single mtDNA compacted by TFAM (Kukat et al., 2011, Kukat et al., 2015). However, we observed that TFAM-containing nucleoids tend to form clustered aggregates in bulbous mitochondria in β-actin-null cells (Figures 3E and 3G, see arrows). We wonder whether this topological change affects TFAM binding on mtDNA. TFAM chromatin immunoprecipitation sequencing (ChIP-seq) analysis on mitochondrial genome shows that TFAM binding was evenly distributed across the whole mtDNA in both WT and KO cells (Figure S4C). Together, these data suggest that β-actin-containing structure is involved in the segregation and distribution of mitochondrial nucleoids, but is not required for the binding of TFAM on mtDNA.

### Mitochondria-Targeted β-Actin Partially Rescues MMP and Increases mtDNA Transcription

**Figure 4:**
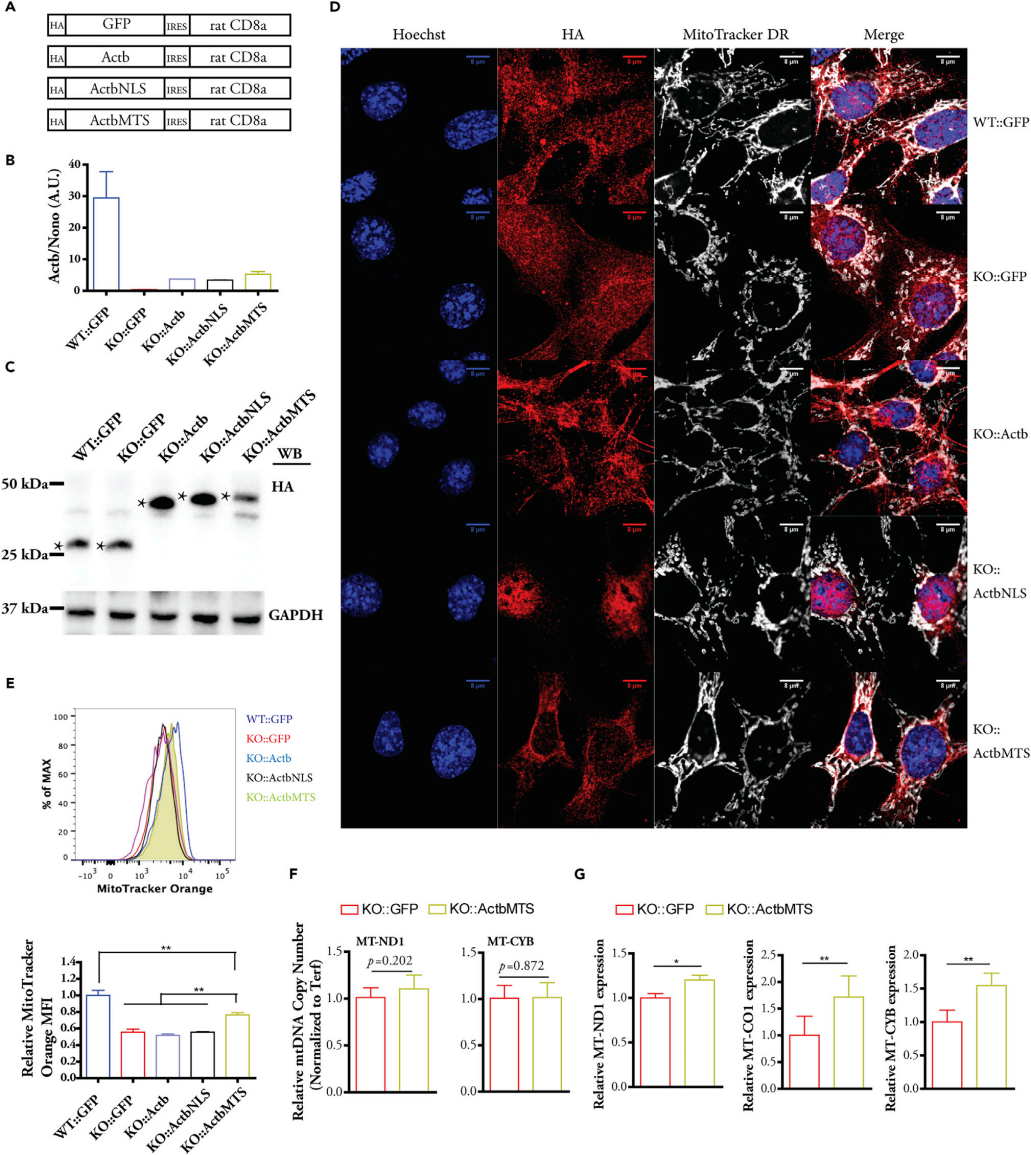
Mitochondria-Targeted β-Actin but Not Nucleus-Targeted β-Actin Shows Rescue Effect on MMP (A) Schematics of retroviral constructs used for re-introducing GFP, β-actin (Actb), β-actin with NLS (ActbNLS), and β-actin with MTS (ActbMTS) into KO (β-actin−/−) MEFs. (B) qPCR quantification of relative expression level of β-actin mRNA. WT::GFP represents WT cells transduced with retrovirus carrying GFP; n = 3 biological replicates. (C) Western blot of protein levels of HA-tagged GFP and β-actin. The HA-GFP (first two lanes), HA-tagged β-actin, β-actin with NLS, and β-actin with MTS are indicated by asterisk, with expected molecular weights. (D) Confocal images of localization of HA-tagged GFP, β-actin, β-actin with NLS, and β-actin with MTS. Scale bar, 8 μm. (E) FACS analysis of MMP stained by MitoTracker Orange; n = 4 independent experiments. One-way analysis of variance (ANOVA) with Tukey's post hoc test: mean ± SEM, **p ≤ 0.01). SEM, standard error of the mean. (F) mtDNA level comparison between KO::GFP and KO::ActbMTS cells; n = 3 biological replicates, Student's t test. (G) Transcript level of MT-ND1, MT-CYB, and MT-CO1 between KO::GFP and KO::ActbMTS cells; n = 3 independent experiments; Student's t test: mean ± SEM *p ≤ 0.05, **p ≤ 0.01. See also Figure S5.

To demonstrate a functional role for the mitochondrial β-actin pool, we used a retroviral system to re-introduce into KO cells WT β-actin, β-actin with NLS, and β-actin with cytochrome c oxidase subunit 4 (COX4) mitochondria-targeting sequence (MTS) (Chatterjee et al., 2016). A retroviral system with rat CD8a extracellular domain as a transduction marker was used to generate cells stably expressing different constructs (Xie et al., 2015). A GFP construct was used as control for viral transduction (Figures 4A and S5A). Cells expressing the transduction marker rCD8a (cell surface marker used for sorting) were sorted by FACS, and about 90% of cells stably expressed rCD8a marker after expansion (Figure S5B, KO::Actb: KO cells transduced with virus carrying *Actb* gene). The re-introduced Actb, ActbNLS, and ActbMTS constructs in KO cells showed comparable messenger RNA (mRNA) expression levels, although at much lower levels when compared with endogenous β-actin in WT cells (Figure 4B). However, the ActbMTS displayed a lower protein level when compared with Actb and ActbNLS constructs (Figure 4C). This may be due to the impaired stability caused by the folding and refolding process during mitochondrial import through the double membrane (Dudek et al., 2013), or due to the mitochondrial protein quality control by proteases (Hamon et al., 2015).

The low expression of re-introduced β-actin was not clearly detected by anti β-actin antibody (data not shown). Instead, we stained the cells with high-affinity anti-hemagglutinin (HA) antibody and observed differential localization patterns (Figure 4D). As expected, we observed actin fiber formation in the cytoplasm of KO::Actb cells and the enrichment of β-actin in the nucleus of KO::ActbNLS cells (Figure 4D). β-Actin with MTS seems to be preferentially localized to the mitochondria, especially those located around the nucleus (Figure 4D). Super-resolution images demonstrated that β-actin with MTS was localized both around and inside the mitochondria (Figure S5C). We analyzed the MMP by MitoTracker Orange staining and found a significant rescue effect of MMP only in KO::ActbMTS cells, when compared with KO::GFP cells (Figure 4E). The same results were seen by TMRE staining analysis (Figure S5D). We found that whereas mtDNA copy number remained unchanged between KO::GFP and KO::ActbMTS (Figure 4F), an increase in the transcript from mtDNA was detected in KO::ActbMTS cells (Figure 4G), suggesting that mitochondrial β-actin can directly regulate mtDNA transcription. Interestingly, upon the expression of ActbMTS we also found a tendency in the up-regulation of nuclear OXPHOS genes (Figure S5E), which further implies a cross talk between mtDNA expression and nuclear transcription of OXPHOS genes.

## Discussion

Our study provides novel and direct evidence supporting the functional localization of β-actin inside mitochondria. Although early studies reported the possible presence of actin in mitochondria, its presence was questioned due to the lack of an MTS in the actin protein and the high potential for contamination in isolated mitochondria (Etoh et al., 1990, Lo et al., 2003). In line with previous observation, our results show the presence of β-actin within mammalian mitochondria and reveal that network-like β-actin structures are interconnected between the cytosol and mitochondria. Recent evidence strongly support the presence of actin in human mitochondria and its tight association with mtDNA (Reyes et al., 2011). It remains to be established whether this association is direct or indirect, occurring through intermediate proteins (Boldogh et al., 2003, Hobbs et al., 2001). Interestingly, in plant cells, electron microscopy with immunogold labeling showed that actin resides in the mitochondrial matrix (Lo et al., 2011). A recent quantitative proteomic analysis of yeast mitochondria also identified yeast actin (ACT1) as a component of mitochondria with high confidence (Morgenstern et al., 2017). Together, these studies also support the presence of actin in the mitochondria of different organisms.

As β-actin and myosin associate with mtDNA (Reyes et al., 2011), it is likely this interconnected actomyosin network functions to support proper mtDNA replication, thereby regulating mtDNA copy number. Interestingly, previous studies reported that actin can be co-purified with mitochondrial nucleoid factors such as TFAM and mitochondrial transcription termination factor 2 (MTERF2) (Kanki et al., 2004, Pellegrini et al., 2009). We observed that in the absence of β-actin-containing network in mitochondria of KO cells, TFAM-based nucleoid clustered to large aggregates. Together, β-actin-containing structure may function as a “mitoskeleton” to physically support TFAM-based nucleoid segregation and distribution. Considering that the ancestral prokaryotes utilize actin-like cytoskeleton in the similar process of plasmid partitioning (Carballido-Lopez, 2006), actin-based mechanism seems to be fundamental for circular DNA segregation.

Our data also demonstrate an essential functional role of β-actin in mitochondrial quality control. Importantly, β-actin is indispensable for maintaining mtDNA transcription and MMP. The absence of β-actin impaired OXPHOS activity and cellular ATP level. It is noteworthy that the recently observed up-regulation of other actin isoforms such as smooth muscle α-actin and cytosolic γ-actin in KO cells (Xie et al., 2018) does not functionally compensate for the loss of β-actin in mitochondrial quality control. In addition, only β-actin but not γ-actin was identified in isolated mtDNA (Reyes et al., 2011), suggesting that the role in maintaining mtDNA transcription, mtDNA copy number, and MMP is specific for β-actin. It is most likely that the lack of mitochondrial pool of β-actin accounts for the mtDNA transcription defects observed in β-actin-null cells because only mitochondria-targeted β-actin shows rescue effects on mtDNA transcription and MMP. In the cell nucleus, β-actin, together with actin-binding proteins, is involved in transcription regulation and chromatin remodeling (Virtanen and Vartiainen, 2017, Visa and Percipalle, 2010). Since mitochondria have their own transcription apparatus, it is plausible to speculate that β-actin interacts with mtDNA-binding proteins to regulate nucleoid topology and affects mtDNA transcription. Further studies are required to address the potential involvement of myosin in mtDNA transcription.

The transcription of nuclear OXPHOS genes seems to be correlated with mtDNA transcription. Without β-actin, mtDNA expression is impaired and nuclear OXPHOS genes are down-regulated. The expression of nuclear OXPHOS genes tend to increase when mitochondria-targeted β-actin is re-introduced to boost mtDNA transcription. Several mito-nuclear communication pathways have been found in both anterograde and retrograde manners under stress conditions and proteostasis regulation (Quiros et al., 2016, Ryan and Hoogenraad, 2007). Although it has been noted that the expression of mtDNA and nuclear OXPHOS genes are correlated (Gagnon et al., 1991), the molecular players mediating the coordination remain to be identified.

One open question is how β-actin is imported into mitochondria. Owing to the unique double membrane structure of mitochondria, mitochondria relies on specialized protein translocases for protein import (Dudek et al., 2013). However, cytosolic proteins can be imported into organelles via uncanonical routes (Lo et al., 2011). Interestingly, a study by Reyes et al. (2011) shows that import of β-actin into isolated mitochondria seems to be dependent on the proton gradient, suggesting an undiscovered route of protein import by mitochondria.

In conclusion, our study unveils a previously unknown role of β-actin inside mitochondria. β-Actin-containing structures inside mitochondria are required for optimal mtDNA transcription and MMP maintenance.

## Methods

All methods can be found in the accompanying Transparent Methods supplemental file.

## Supplemental Information

Document S1. Transparent Methods and Figures S1–S5

Video S1. Cell Morphology Change after CCCP Addition, Related to Figure 1 CCCP was added into culture medium of WT and KO cells at 30 μM. Time lapse images were taken immediately after CCCP addition with the same interval. Time tracker was displayed at the left top corner of the video

Video S2. Mitochondria Morphology of WT and KO MEFs, Related to Figure 1 WT and KO cells were stained with 200 nM MitoTracker Deep Red in culture medium for 30 min. MitoTracker dye was washed and replaced with fresh medium and live cell images of stained mitochondria was captured

Video S3. Mitochondrial Morphology Changes after MPP+ Stress, Related to Figure 1 WT and KO cells were stained with MitoTracker Deep Red in culture medium. After staining, MPP+ was added to the culture medium at 2 mM and live cell images were taken immediately after MPP+ addition
