# Supplementary material for: In Mitochondria β-Actin Regulates mtDNA Transcription and Is Required for Mitochondrial Quality Control
Source: iScience. 2018 May 3;3:226–37. doi: 10.1016/j.isci.2018.04.021 (PMC6137402; doi:10.1016/j.isci.2018.04.021)
Supplement: Document S1. Transparent Methods and Figures S1–S5 [file mmc1.pdf]

ISCI, Volume *3*

Supplemental Information

In Mitochondria β-Actin Regulates mtDNA Transcription and Is Required for Mitochondrial Quality Control

Xin Xie, Tomas Venit, Nizar Drou, and Piergiorgio Percipalle

Supplemental Figures and Legends

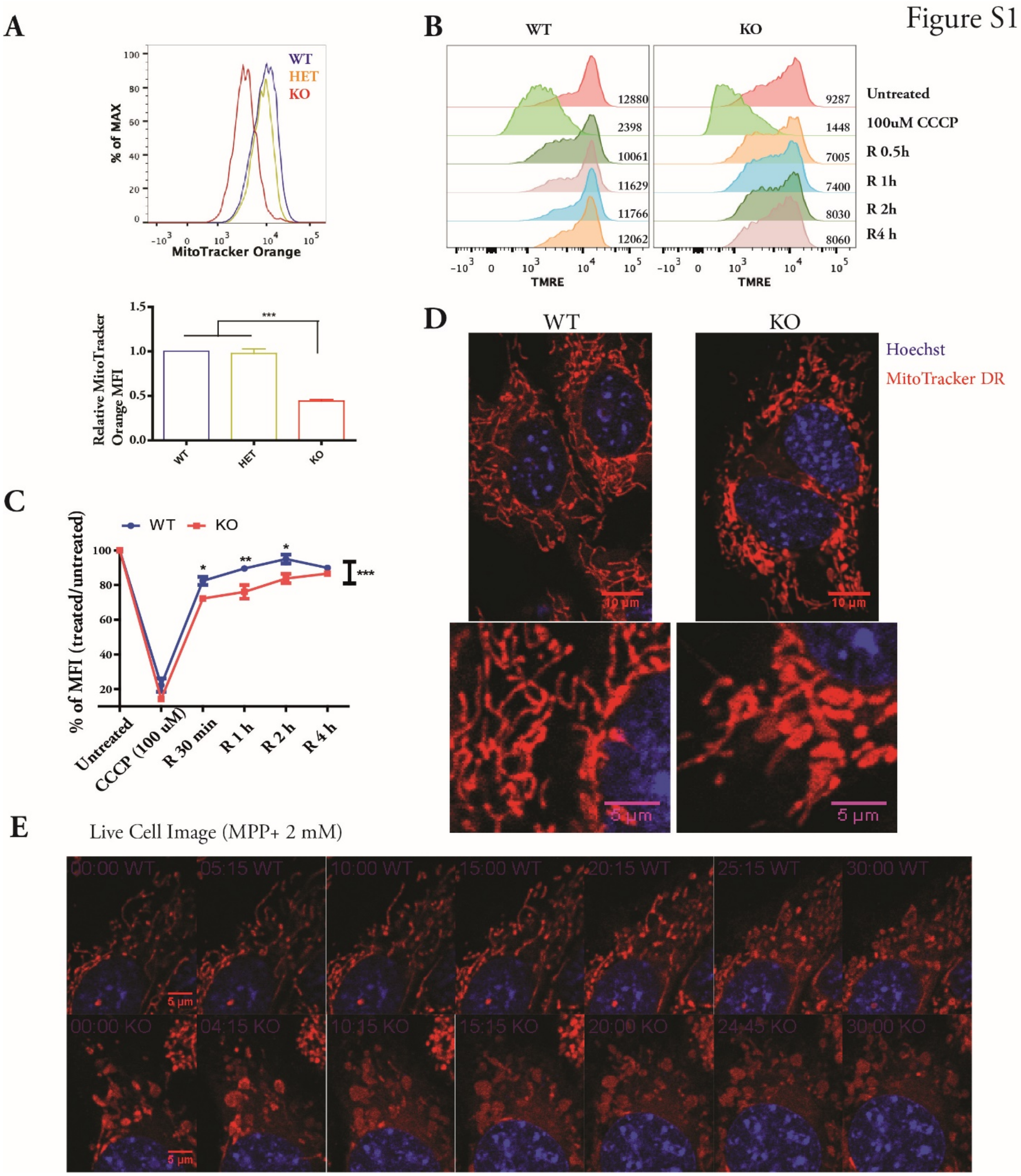

Figure S1. Impaired ability to maintain MMP and altered mitochondira morphology in β-actin null MEFs, related to Figure 1.

(A) FACS analysis of MMP in WT (β-actin+/+), HET (β-actin+/-) and KO (β-actin-/-) MEFs, stained by MitoTracker Orange (n=3). One-way ANOVA: ***: $p<0.001$, Mean ± S.E.M.

(B-C) WT and KO MEFs were depolarized by 100 μM CCCP for 2 h. Then CCCP-contained medium was replaced with fresh medium. The MMP changes at each time point was determined by TMRE staining in (B). The mean TMRE fluorescence intensity of untreated WT and KO cells were set as 100 and the MMP at each time point was normalized to untreated sample in (C). Data are summary of 3 biological replicates. Two-way ANOVA: *: $p<0.05$; **: $p<0.01$; ***: $p<0.001$. Mean ± S.E.M.

(D) Confocal images of live cell mitochondria staining by MitoTracker Deep Red.

(E) Live cells stained with MitoTracker Deep Red were treated with MPP+. A serious of confocal images were taken immediately after MPP+ addition. The corresponding time points of each image were shown on the top left.

Figure S2

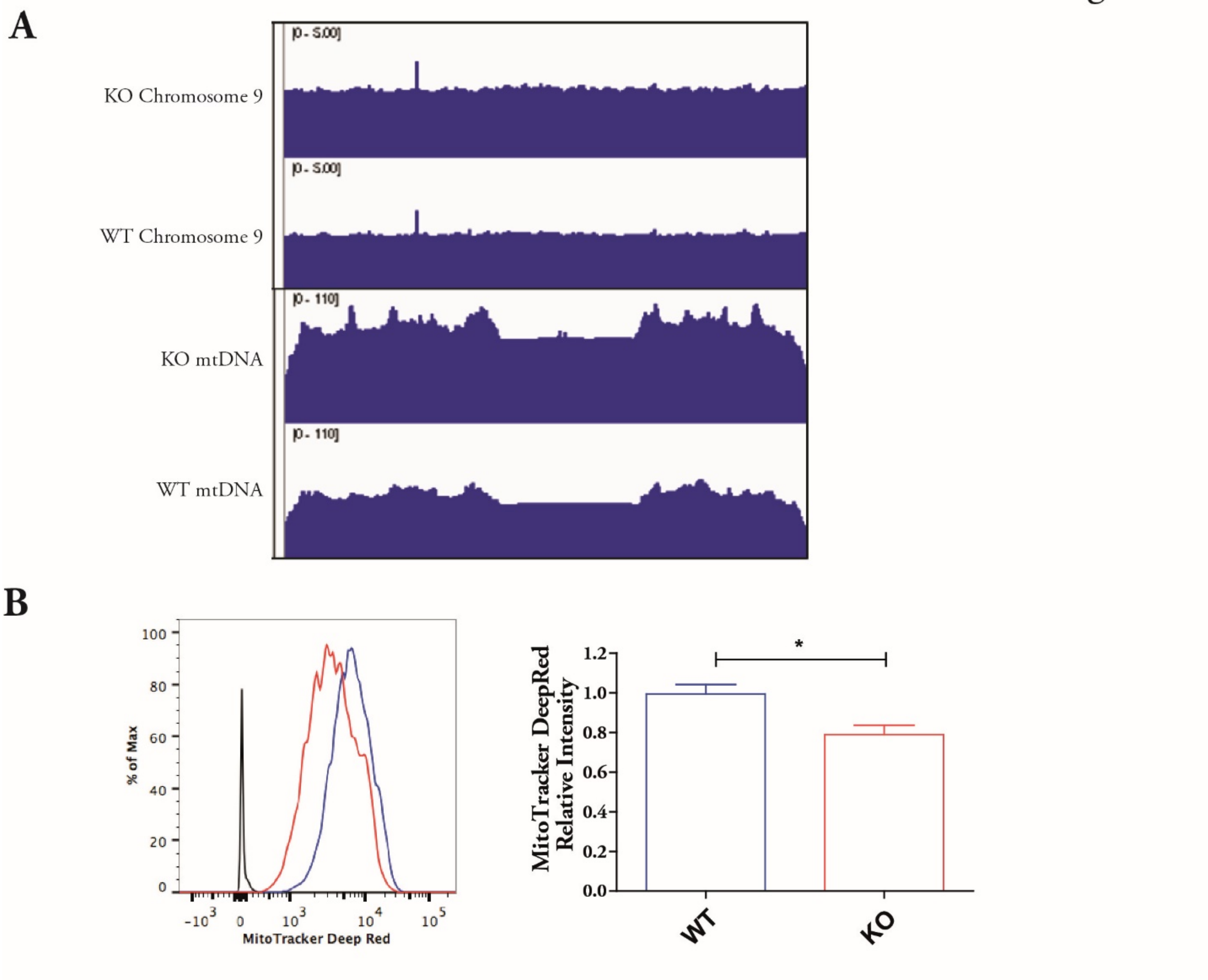
A
[0 - 5.00]
KO Chromosome 9
[0 - 5.00]
WT Chromosome 9
[0 - 110]
KO mtDNA
[0 - 110]
WT mtDNA
B
100
80
60
40
20
0
% of Max
-10^3
0
10^3
10^4
10^5
MitoTracker Deep Red
MitoTracker DeepRed Relative Intensity
1.2
1.0
0.8
0.6
0.4
0.2
0.0
*
WT
KO

Figure S2. Increased mtDNA copy number but reduced mitochondrial mass in KO cells, related to Figure 2.

(A) Total DNA isolated from cells were subjected to DNA sequencing. Normalized sequencing reads shows the relatively higher level of mtDNA in KO cells when compared to WT cells, while the nuclear reads level are the same between WT and KO cells.

(B) FACS estimate of mitochondrial mass by MitoTracker Deep Red staining. Mean fluorescence intensity (MFI) of MitoTracker Deep Red staining was summarized from 3 biological replicates. Student's t Test, * $P<0.05$. Mean ± S.E.M.

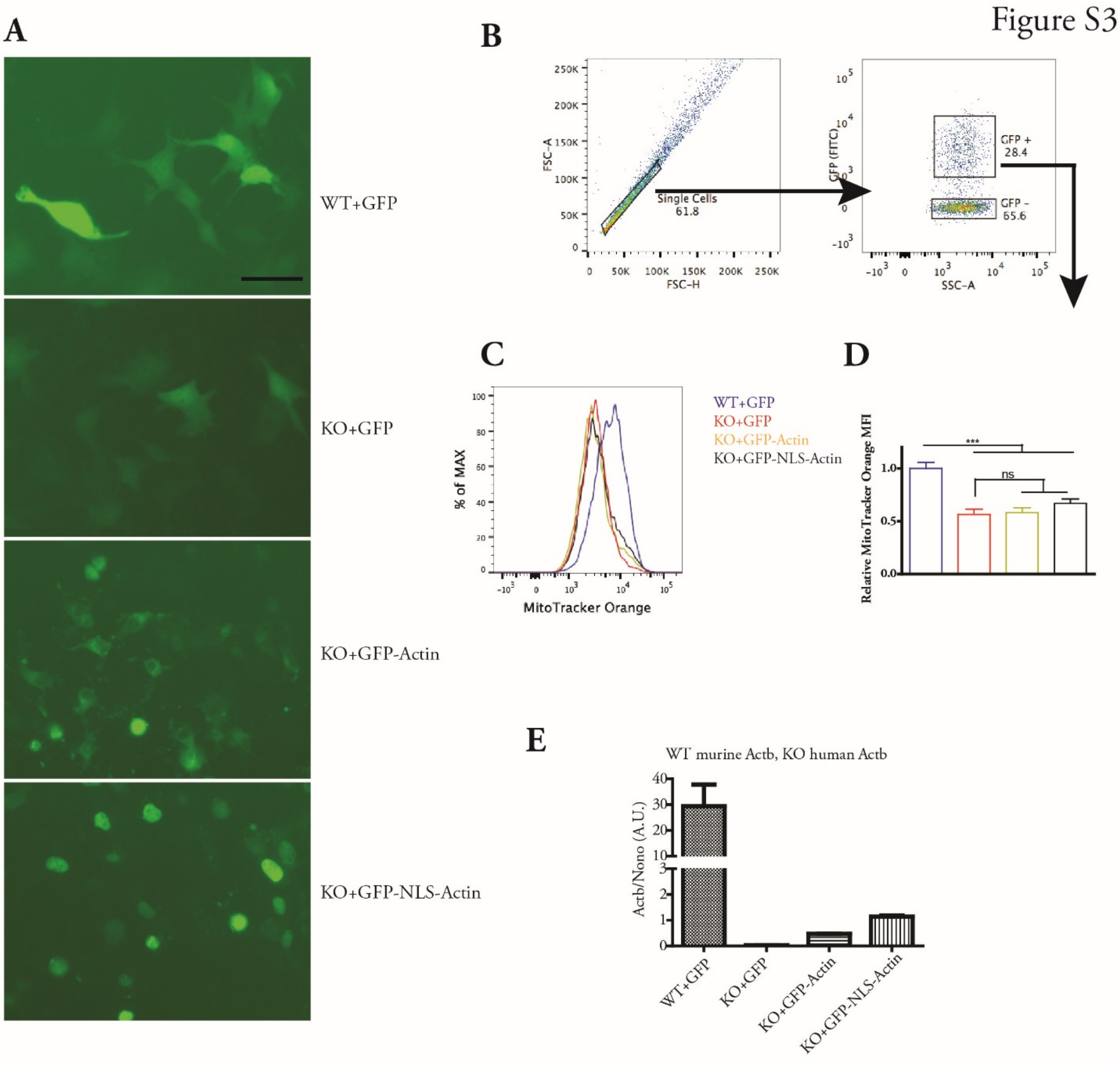
Figure S3
A
WT+GFP
KO+GFP
KO+GFP-Actin
KO+GFP-NLS-Actin
B
FSC-A
FSC-H
Single Cells
61.8
GFP (FITC)
SSC-A
GFP +
28.4
GFP -
65.6
C
% of MAX
MitoTracker Orange
WT+GFP
KO+GFP
KO+GFP-Actin
KO+GFP-NLS-Actin
D
Relative MitoTracker Orange MFI
***
ns
E
WT murine Actb, KO human Actb
Actb/Nono (A.U.)
WT+GFP
KO+GFP
KO+GFP-Actin
KO+GFP-NLS-Actin

Figure S3. Transient expression of actin or NLS-targeted actin failed to rescue MMP in KO cells, related to Figure 3.

(A) Images showing the distribution of GFP signal in GFP, GFP-actin and GFP-NLS-Actin transfected cells.

(B-D) Cells were transfected with GFP, GFP-actin and GFP-NLS-Actin construct. 48 h post-transfection, cells were stained with MitoTracker Orange and analyzed by FACS. (B). Gating of GFP+ cells were shown. (C). Overlaid histograms showing GFP+ population of each samples in terms of the MitoTracker Orange staining intensity. (D). Relative of MitoTracker Orange MFI, data are summary of 3 biological replicates, representative of 2 independent experiment. One-way ANOVA: ***: $p<0.001$. Mean ± S.E.M.

(E) Relative β-actin mRNA levels. In WT and KO cells with GFP, endogenous β-actin was determined using primer specific for murine β-actin. In KO+GFP-Actin and KO+GFP-NLS-Actin cells, introduced β-actin from human origin was determined using primer specific for human β-actin. Data were normalized to endogenous *Nono* gene. Data show the triplicate measurement of sorted GFP+ cells. Mean ± S.E.M.

Figure S4

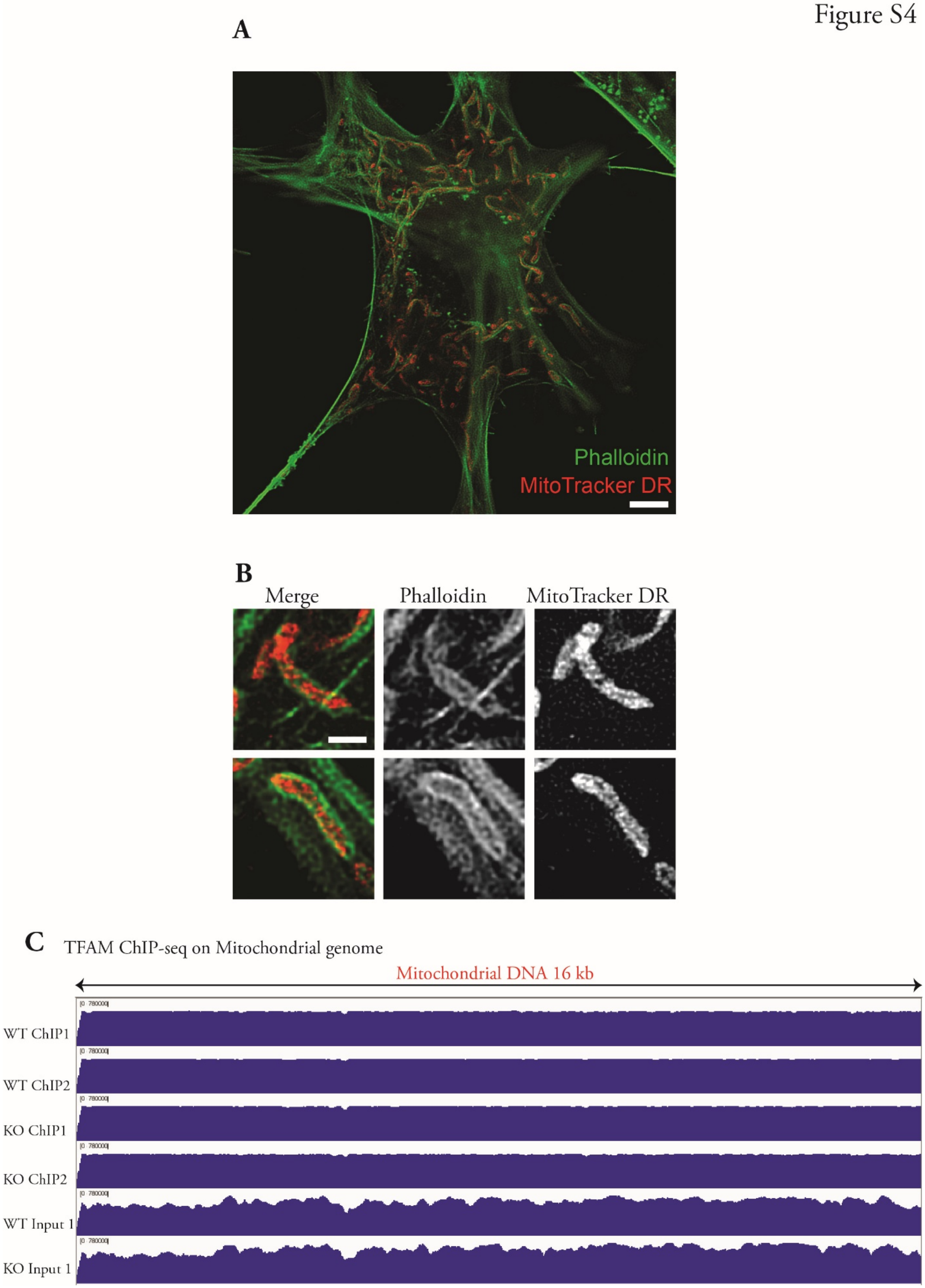
A
Phalloidin
MitoTracker DR
B
Merge
Phalloidin
MitoTracker DR
C
TFAM ChIP-seq on Mitochondrial genome
Mitochondrial DNA 16 kb
WT ChIP1
WT ChIP2
KO ChIP1
KO ChIP2
WT Input 1
KO Input 1

Figure S4. Super-resolution image of Mitochondria and Phalloidin co-staining, related to Figure 3.

(A) WT MEFs were stained with MitoTracker Deep Red and Phalloidin-iFluor 555 and visualized using STED microscope. Scale Bar: 5 µM.

(B) Insets from (A) showing the strong phalloidin staining surrounding mitochondria and weak phalloidin staining within mitochondria. Scale Bar: 1 µM.

(C) TFAM ChIP-seq analysis on mitochondrial genome. Two biological replicates of TFAM ChIP-seq and one input of each cell type was shown. TFAM ChIP-seq signals in WT and KO cells show even distribution across the whole mitochondrial genome. Y-axis of each track shows the value of RPKM (Reads Per Kilobase per Million mapped reads).

Figure S5

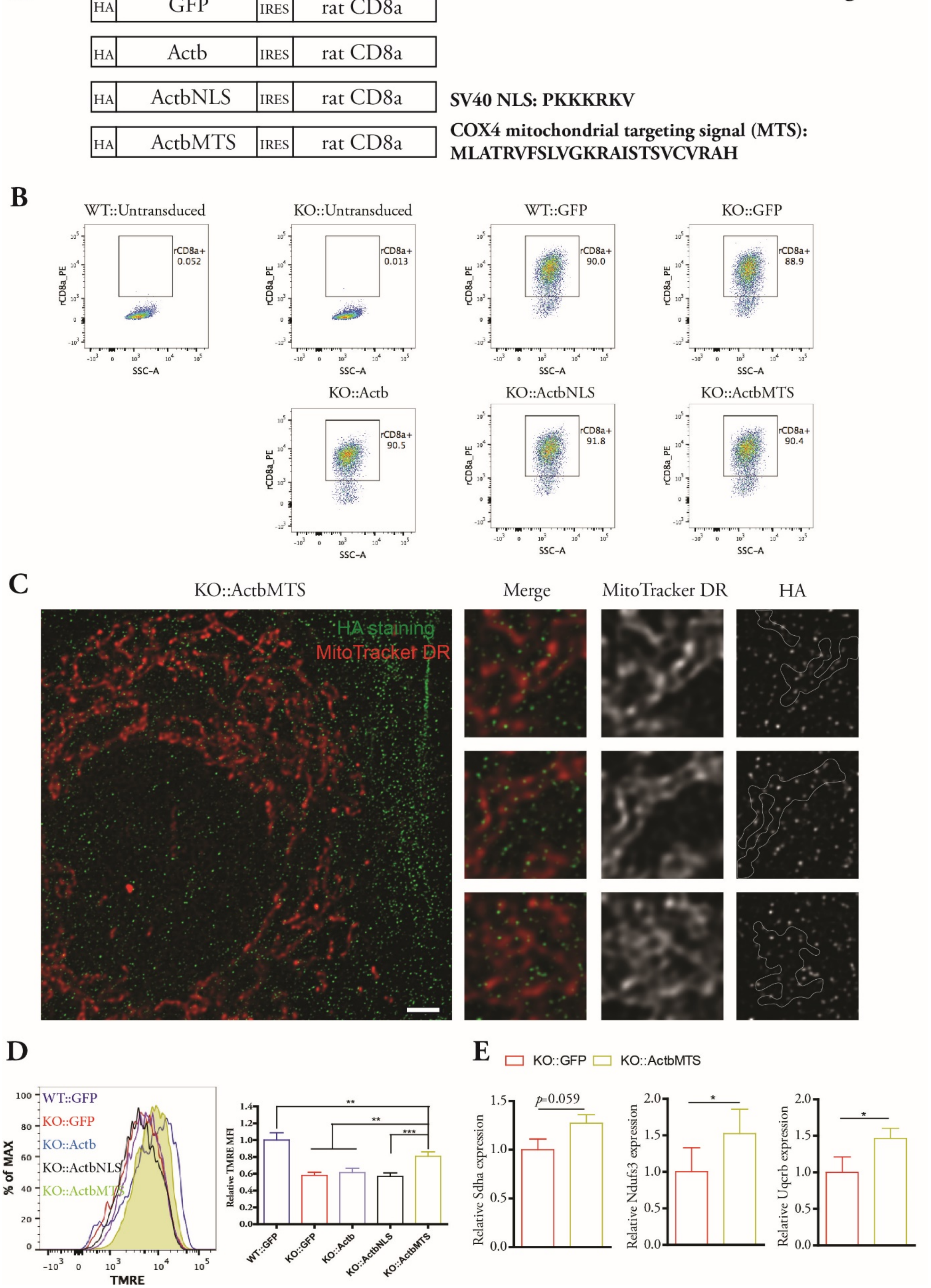
A
HA
GFP
IRES
rat CD8a
Actb
ActbNLS
ActbMTS
SV40 NLS: PKKKRKV
COX4 mitochondrial targeting signal (MTS):
MLATRVFSLVGKRAISTSVCVRAH
B
WT::Untransduced
KO::Untransduced
WT::GFP
KO::GFP
KO::Actb
KO::ActbNLS
KO::ActbMTS
rCD8a+
0.052
0.013
90.0
88.9
90.5
91.8
90.4
rCD8a_PE
SSC-A
C
KO::ActbMTS
HA staining
MitoTracker DR
Merge
MitoTracker DR
HA
D
WT::GFP
KO::GFP
KO::Actb
KO::ActbNLS
KO::ActbMTS
% of MAX
TMRE
Relative TMRE MFI
**
**
***
E
KO::GFP
KO::ActbMTS
p=0.059
*
*
Relative Sdha expression
Relative Ndufs3 expression
Relative Uqcrb expression

Figure S5. Mitochondria-targeted β-actin partially rescue MMP in KO cells, related to Figure 4.

(A) Schematics of retroviral construct used for reintroducing Actb gene, Actb with SV40 NLS (nucleus localization signal) or Actb with Cox4 MTS (mitochondrial targeting signal) into KO cells. rCD8a was co-expressed as a surface marker for sorting. GFP was used as control.

(B) Cells transduced with corresponding virus (e.g. KO::GFP, KO cells transduced with virus carrying GFP). Transduced cells were stained with anti-rCD8a-PE antibody and sorted for PE+ population. Dot plots show the percentage of rCD8a+ cells after sorting and cell expansion.

(C) STED microscope image showing the localization of HA-tagged β-actin with MTS (ActbMTS) inside mitochondria. Scale bar: 3 µm. Several insets of MitoTracker DR and HA staining examples were shown in right panel.

(D) Cells in were stained with TMRE and analyzed by FACS. The relative mean TMRE fluorescence intensity were shown in histogram. Data is the summary of 3 independent experiments. One-way ANOVA: **: $p<0.01$, ***: $p<0.001$. Mean ± S.E.M.

(E) qPCR quantification of relative nuclear OXPHOS genes expression in KO::GFP and KO::ActbMTS cells. Data is the summary of 3 independent experiments. Student's t test: *: $p<0.05$. Mean ± S.E.M.

Transparent Methods

Antibodies and reagents:

Antibody of HA (ab9110) and Phalloidin-iFluor 555 reagent was from Abcam. Antibody against β-actin (clone AC-74), Carbonyl cyanide 3-chlorophenylhydrazone (CCCP, C2759), MPP^+ iodide (D048), Tetramethylrhodamine ethyl ester perchlorate (TMRE, 87917), Rotenone (R8875), 2-Thenoyltrifluoroacetone (TTFA, T27006), Antimycin A (A8674), Potassium cyanide (KCN, 60178), N,N′-Dicyclohexylcarbodiimide (DCCD, 36650), polybrene (H9268), DMEM medium high glucose (D5671), Fetal Bovine Serum (F0804), Penicillin-Streptomycin (P0781), 2X Laemmli Sample buffer (S3401) were from Sigma-Aldrich. Antibodies of mouse IgG Dylight 550 (84540), rabbit IgG Dylight 550 (84541), MitoTracker™ Orange CMTMRos (M7510), MitoTracker™ Deep Red FM (M22426), Hoechst 43222 (H1399), Click-iT™ Nascent RNA Capture Kit (C10365), Maxima SYBR Green qPCR Master Mix (K0252), RevertAid First Strand cDNA Synthesis Kit (K1622), DNA-free™ DNA Removal Kit (AM1906), Mitochondria Isolation Kit (89874), Pierce™ ECL Western Blotting Substrate (32106), Pierce™ Protein Assay Kit (23225), ATP Determination Kit (A22066), PureLink Genomic DNA Mini Kit (K182001), TRIzol® Reagent (15596-018) and Lipofectamine™ 3000 Transfection Reagent (L3000015) were purchased from Thermo Fisher Scientific. Anti-rat CD8a-PE (201706) antibody was purchased from Biolegend. Anti-GAPDH-HRP (HRP-60004) was from ProSci. MitoCheck® Complex II/III Activity Assay Kit (700950) was from Cayman Chemical. Qiaquick PCR Purification Kit (28106) and RNeasy Mini Kit (74106) were purchased from Qiagen. Anti-TFAM (ABE483) and Magna ChIP Protein A+G Magnetic Beads (16-663) were from Merk Millipore. The concentration of the compounds and dilution of antibodies are indicated in each experiment respectively.

Cell culture:

The β-actin$^{+/+}$ MEFs (WT), β-actin$^{+/-}$ MEFs (HET) and β-actin$^{-/-}$ MEFs (KO), and mouse endothelial cell line C166 (ATCC) were maintained and cultured with Dulbecco's modified Eagle medium (DMEM) with high glucose (Sigma), 10% fetal bovine serum (Sigma) and 100 units/mL penicillin and 100 µg/mL streptomycin (Sigma), in a humidified incubator with 5% CO^2 at 37 ^oC.

Mitochondrial analysis using high-content screening (HCS) Platform:

MEFs were cultured in 96-well back, clear bottom plate (Corning), at density of 5000 cells/well. After 24 h culture, cells were stained with 100 nM MitoTracker Orange CMTMRos for 20 min. Stained cells were washed twice with PBS and fixed by 3.7% formaldehyde for 15 min. After 2 times PBS wash, cells were further stained with Hoechst 43222 (1:6000) for 15 min. After 2 times wash with PBS, stained cells in plate were scanned via Cellomics ArrayScan™ XTI High Content Analysis (HCS) platform (Thermo Fisher Scientific), with a 20x Objective. Compartment Analysis Bio Application software (Cellomics) was applied to quantitatively analyze the MitoTracker staining spots in the simulated cytoplasm of individual cells based on nuclear Hoechst staining. For each experiment, at least 500 valid single cells per culture well were quantified and at least 10 independent culture wells (10 biological replicates) were analyzed.

Flow cytometry analysis:

To compare the membrane potential between WT and KO cells, cells at 70% confluence in 24 or 6 well plate were incubated with medium containing 100 nM MitoTracker Orange CMTMRos, or 0.2 to 2.0 µM TMRE for 20 min. For the assessment of mitochondrial mass, cells were stained with 200 nM MitoTracker™ Deep Red FM for 30 min. After staining, cells were washed 2 times with PBS. Trypsinized cells were re-suspended in PBS, following by immediate analysis via flow cytometer BD FACSAria III.

For the assessment of chemical inhibitors on membrane potential changes, cells were incubated with medium containing CCCP (30 µM), MPP+ (2mM) for 1h, Rotenone (15 µM), TTFA (800 µM) Antimycin A (30 µM), KCN (2 mM) and DCCD (150 µM) for 20 min. Cells with or without treatment were stained with 1 µM TMRE for 20 min. After 2 times washes with PBS, trypsinized cells were re-suspended in PBS and immediately analyzed by flow cytometer BD FACSAria III.

For the analysis of mitochondrial membrane potential recovery after CCCP treatment, cells were treated with 100 µM CCCP for 2 h and cells were washes once with fresh medium. Fresh medium was added to allow the cells to recover from CCCP-induced membrane potential loss. Cells at different time points of recovery were stained with 1 µM TMRE for 20 min and then analyzed by FACS.

For all samples, at least 10,000 cell events were recorded. Data from flow cytometer BD FACSAria III were analyzed using Flow Jo software.

RNA-Seq analysis:

Total RNA was extracted from 70% confluent cells using TRI Reagent according to the manufacturer protocol (Sigma-Aldrich). Quality of total RNA were evaluated at SciLIfe lab (Stockholm, Sweden) using Qubit and Bioanalyzer respectively. 1 µg total RNA of Samples that passed the QC assessment were used for library construction using TruSeq Stranded mRNA Library Prep Kit (Illumina) according to the manufacturer protocol. Deep sequencing was performed at SciLife Laboratory, the National Genomics Infrastructure, NGI, Karolinska Institute, Stockholm. Illumina HiSeq 2000 sequencer was used to produce 50 bp single-end reads, following manufacturers' protocols.

The sequencing data was processed through the standard RNAseq analysis pipeline at NYUAD. Sequenced reads were trimmed for adaptor sequence, and masked for low-complexity or low-quality sequence, then mapped to the Mus musculus GRCm38.p4 genome using tophat2 v2.1.0 with the parameters "–no-novel-junctions" and "–G" when specifying the genome file. Cufflinks v2.2.1 was used to derive FPKM (Fragments Per Kilobase of transcript per Million mapped reads). Differential gene expression was analyzed by Cuffdiff. RNA-seq data was deposited in GEO repository and the GEO accession number is GSE95830.

Immunofluorescence, live cell imaging and super-resolution microscopy:

Cells grown on glass cover slip were stained with 100 nM MitoTracker Orange CMTMRos, or 200 nM MitoTracker™ Deep Red FM for 20 min. Stained cells were then washed 2 times with fresh medium and then cultured in fresh medium for 1 h before being fixed by 70% cold ethanol for antibody staining. For phalloidin staining, cells were fixed with 4% formaldehyde. Fixed cells were permeabilized with 0.5% Triton X-100 in PBS for 15 min. After blocking by 1% BSA for 1 h, cells were stained with primary antibodies against HA (1:200), TFAM (1:200) or β-actin (1:150), or Phalloidin iFluor 555 reagent (1:1000) for 2 h. The cells were washed 3 times with TBST buffer. The antibody-stained cells further stained with corresponding dylight 550-conjugated secondary antibody (1:1000) and Hoechst 43222 (1:6000) for 1 h. The cover slip was mounted to glass slide using Molecular Probes ProLong® Gold anti-fade mounting media (Invitrogen). Stained cells were observed using Olympus FV1000 confocal microscope.

For live cell imaging of mitochondria, cells grown on glass-bottom CELLview culture dish (Greiner Bio One) were stained with 200 nM MitoTracker™ Deep Red FM for 20 min. After 2 times washes with fresh medium, cells were cultured in incubator for 2 h before imaging. Imaging of mitochondria was performed in a temperature and CO^2 controlled chamber that is connected with Olympus FV1000 confocal microscope system. A time series of confocal images of untreated cells or cells immediately after treatment were captured. Confocal images were captured with 63× immersion oil objective lens, NA 1.4 and a LAS AF software.

Super-resolution images were acquired using Leica TCS SP8 STED 3X microscope equipped with HyD SMD2 detector and Leica HCPL APO CS2 100x/1.51 oil objective. Software Leica application SuiteX was used for capturing and analysis of the images. Huygens Professional software was used for deconvolution. Final data were analyzed and processed by using Fiji software.

TFAM ChIP-seq and analysis:

For TFAM ChIP-seq, chromatin cross-linked *in vivo* by 1% formaldehyde was quenched by 0.125 M Glycine and fragmented using S220 Focused-ultrasonicator (Covaris). 50 ug total fragmented chromatin were mixed with 7 ug anti-TFAM (ABE483) antibody overnight with rotation at 4 C^o. The chromatin-antibody complex were then precipitated with Magna ChIP protein A/G magnetic beads. After washes with low salt wash buffer (20 mM Tris, 2 mM EDTA, 50 mM NaCl, 0.01% SDS and 1% SDS), high salt wash buffer (same composition, 250 mM NaCl) and LiCL-wash buffer (10 mM Tris, 1 mM EDTA, 250 mM LiCl, 1% NP40 and 1% sodium deoxycholate), precipitated chromatin was eluted in 200 µL of elution buffer (10 µL 20% SDS, 20 µL 1M $NaHCO_3$ and 170 µL dH_2O).

Reverse cross-linking was done by adding 8 uL 5M NaCl into the eluted solution, with overnight incubation at 65 C^o. After reversing the cross-linking, 1 uL 10 mg/ml RNase A was added, with

30 min incubation at 37°C. Then 4 µL 0.5 M EDTA, 8 µL 1M Tris-HCl and 1 µL 20 mg/mL Proteinase K was added to digest the proteins for 2h at 42°C. The released DNA was purified using QIAquick PCR purification kit (Qiagen). Two biological replicates of ChIP-purified DNA and fragmented total DNA, were used for library preparation using TruSeq Nano DNA Library Prep Kit (Illumina). Deep-sequencing was performed using Illumina HiSEq 2500 sequencing platform to generate 100 bp pair-end reads (New York University Abu Dhabi Sequencing Center).

ChIP-seq data were deposited in GEO database: accession number GSE109532. For analysis, the raw reads were quality-trimmed using Trimmomatic package, and aligned to mitochondrial genome based on mouse reference genome (GRCm38.p4) using Burrows-Wheeler Aligner BWA-MEM 9. Picard tools was used to clean, sort and deduplicate the aligned reads. The processed alignments were normalized in RPKM (reads per kilobase per million mapped reads) with DeepTools2 bamCoverage function.

Western Blotting:

Cell lysate was collected by lysing cells in RIPA buffer and protein concentration was determined by BCA protein assay kit (Thermo Fisher Scientific). Samples were mixed with 2X Laemmli Sample buffer (Sigma) and heated at 95 ^{o}C for 5 min. Immunoblotting was performed using anti-HA (1:1000), anti-GAPDH-HRP (1:1000) and goat anti-rabbit IgG HRP (1:2500) antibodies. Protein bands were developed using Pierce ECL western blotting substrate (Thermo Fisher Sceintific), and analyzed by ChemiDoc MP Imaging system (Bio-Rad).

RNA isolation and qPCR:

Total RNA was extracted using RNeasy Mini Kit (Qiagen) according to the manufacturer's instruction. This is followed by DNA removal with DNA-free™ DNA Removal Kit (Themo Fisher Scientific). 500 ng to 1 µg DNase-treated RNA was then reverse transcribed to cDNA using RevertAid First Strand cDNA synthesis Kit (Thermo Fisher Scientific). Quantitative real-time PCR was performed using Maxima SYBR Green qPCR Mix (Thermo Fisher Scientific) on Stratagene 3005 qPCR system (Agilent Technology). All the target gene expression level was normalized to the expression of *Nono* reference gene. Primers of qPCR was listed below:

Gene	Forward primer: 5' – 3'	Reverse primer: 5' – 3'
Actb	TATCGCTGCGCTGGTCG	CCCACGATGGAGGGGAATAC
12S rRNA	TGGTAAATTTCGTGCCAGCCA	AGTTGACACGTTTTACGCCGA
MT-CO1	TTGCAACCCTACACGGAGGT	TCCGGTTAGACCACCAACTGT
MT-ND1	TCGACCTGACAGAAGGAGAATCA	GGGCCGGCTGCGTATT
MT-CYB	AGACAAAGCCACCTTGACCC	GATTGCTAGGGCCGCGATAA
SDHA	GGAACACTCCAAAAACAGACCT	CCACCACTGGGTATTGAGTAGAA
NDUFS3	TGGCAGCACGTAAGAAGGG	CTTGGGTAAGATTTCAGCCACAT
UQCRB	GGCCGATCTGCTGTTTCAG	CATCTCGCATTAACCCCAGTT
Nono	GCCAGAATGAAGGCTTGACTAT	TATCAGGGGGAAGATTGCCCA

RNA synthesis and decay experiment:

Click-iT® Nascent RNA Capture Kit (Thermo Fisher Scientific) was used in this experiment according to the manufacturer's protocol, with some modifications. MEFs cells were tagged with 0.15 mM 5-Ethynyl uridine (EU) as below:

For RNA synthesis analysis, cells were labeled with EU containing growth media for either 2 h (EU 2h) or 4 h (EU 4h). The relative newly synthesized RNA was calculated for 4 h over 2 h pulse labeling duration.

In RNA decay analysis, cells were labeled with EU containing growth media for 2 h (EU 2h). Then cells were washed with fresh culture growth media and cultured for 20 h (EU 2h D20h) in the fresh media without EU.

Total RNA from each sample was harvested using TRIzol® Reagent (Thermo Fisher Scientific). 5 µg total RNA was used in Cu (I) mediated click reaction according to manufacturer's instruction, during which newly synthesized EU-labeled RNA was tagged with biotin. The RNA samples were harvested with TRIzol® Reagent. 150 ng of biotin-tagged RNA were mixed with MYOne T1 Streptavidin magnetic beads to pull down the biotinylated RNA. After sequential washes using Click-iT wash buffer I and wash buffer II, captured RNA on the beads was converted to cDNA by RevertAid First Strand cDNA synthesis Kit (Thermo Fisher Scientific). qPCR quantification was performed in the same manner as described in RNA isolation and qPCR section.

mtDNA copy number quantification:

Total mitochondrial and nuclear genomic DNA was collected using PureLink Genomic DNA Mini Kit (Thermo Fisher Scientific), with protease and RNase treatment according to the manufacturer's instructions. The purified total genomic DNA was quantified by Quantitative real-time PCR, and the mtDNA level was normalized to that of nuclear *Terf* gene. Primers were listed below:

Gene	Forward Primer: 5' – 3'	Reverse primer: 5' – 3'
Terf	CTAGCTCATGTGTCAAGACCCTCTT	GCCAGCACGTTTCTCTCGTT
MT-ND1	ACACTTATTACAACCCAAGAACACAT	TCATATTATGGCTATGGGTCAGG
MT-CYB	AGCCACCTTGACCCGATTCT	CGTGGAGGAAGAGGAGGTGA
MT-ATP6	GCAGTCCGGCTTACAGCTAA	GGTAGCTGTTGGTGGGCTAA

For DNA deep sequencing, total DNA from 1% formaldehyde-fixed MEFs was subject to fragmentation using S220 Focused-ultrasonicator (Covaris). DNA library was prepared by TruSeq Nano DNA Library Prep Kit (Illumina), and then sequenced using Illumina HiSEq 2500 sequencing platform (New York University Abu Dhabi Sequencing Center). The normalized reads (Reads per Kilobase of sequence range per Million mapped reads) were used to compare the relative level of nuclear DNA and mtDNA.

Mitochondrial Complex II/III activity assay:

Mitochondria from $2x10^7$ MEFs were purified using Mitochondria Isolation Kit for Cultured Cells (Thermo Fisher Scientific) according to the manufacturer's instruction. The complex II/III activity of freshly isolated mitochondria were determined using MitoCheck® Complex II/III Activity Assay Kit (Cayman Chemical). Briefly, 20 µL mitochondria were mixed with 958 µL Complex III activity assay buffer, 2 µL of 1mM Rotenone and 20 µL of 100 mM KCN as reaction A. Reaction B contains 607 µL Complex III activity assay buffer, 8 µL Succinate reagent and 60 µL Cytochrome c reagent. 50 µL of reaction A was mixed with 20 µL Complex III activity assay buffer and 30 µL reaction B in a 96 well plate. The plate was immediately measured for a kinetic at absorbance 550 nm for 15 min with an interval of 20 seconds at room temperature, using Synergy H1 microplate reader (BioTek). Data was plotted as absorbance (y axis) versus time (x-axis) and the slope of linear portion was the reaction rate, which stands for Complex II/III activity.

ATP determination:

1.5 x 10^4 MEFs in 96 well plate were lysed with 150 uL NP-40 lysis buffer (150 mM NaCl, 1.0% NP-40 and 50 mM Tris-Cl, pH8.0) on ice for 10 min. After mixing by pipetting up and down, the ATP level was determined using ATP Determination Kit (Thermo Fisher Scientific) according to the manufacturer's instructions. Briefly, reaction solution containing 1X reaction buffer, 1 mM DTT, 0.5 mM D-luciferin, 1.25 µg/mLfirefly luciferase was prepared. 10 µL cell lysate was mixed with 100 µL reaction solution in white 96 well plate and then measured for luminescence via filter set 1 (band pass 528/20) of Synergy H1 microplate reader (BioTek). Blank reaction and standard ATP solution was included as controls.

Cell transfection:

MEFs in 6 well plates were transfected with 3 µg GFP-Actin, GFP-NLS-Actin and M6P-GFP plasmids using Lipofectamine™ 3000 Transfection Reagent (Thermo Fisher Scientific). 48 h post-transfection, the expression of introduced proteins were imaged by EVOS Cell Imaging Systems (Thermo Fisher Scientific) and mitochondria membrane potential was analyzed by FACS.

Retrovirus production and cells transduction:

Retrovirus vector, virus preparation and cell transduction were performed as described previously (Xie et al., 2015). Mouse β-actin open reading frame was cloned from the cDNA of WT MEF and ligated into a retrovector with rCD8a as transduction marker. An SV40 nucleus localization signal (PKKKRKV) or a COX4 mitochondria targeting signal (MLATRVFSLVGKRAISTSVCVRAH) (Chatterjee et al., 2016) was added to the C-terminal of β-actin. Virus supernatant was produced by transfecting 293FT cells in 10 cm dishes with 12 µg of retroviral vector and 12 µg pCL-Eco packaging plasmid (Novus Biologicals) using the Calcium-phosphate precipitation method. Supernatant of transfected 293FT cells was collected at 48 h and 72 h post-transfection and was snap-frozen in liquid nitrogen. For retroviral transduction, MEF cells in 96 well plate were incubated with 100 µL medium and 100 µL viral supernatant, in the presence of 8 µg/mL polybrene. The cell culture were then centrifuged at 2500 rpm for 1.5 h at 32°C to increase the transduction efficiency, and then transferred to incubator. After 12 h incubation, virus-containing medium was

replaced with fresh medium. Cells after expansion were stained with anti-rat CD8a-PE antibody (1:200 in PBS with 5 mM EDTA) for 15 min. Stably transduced rCD8a+ cells were sorted by FACS (BD FACSARIA III) and the sorted cells were further expanded in DMEM with 20% FBS.

Statistics:

All the data values were expressed as means ± SEM. The number of experiments or biological replicates are indicated in the respective figure legends. For direct comparison between two groups, Student's t test was applied. For the comparison of multiple groups, one-way or two-way ANOVA analysis were used, with Tukey's multiple comparison as post hoc test. *P* value less than 0.05 was considered to be statistically significant.
